# Is poor sleep quality associated with a deterioration in postural control?

**DOI:** 10.5935/1984-0063.20200061

**Published:** 2021

**Authors:** Tarushi Tanwar, Zubia Veqar, Amer K. Ghrouz, David Warren Spence, Seithikurippu R. Pandi-Perumal

**Affiliations:** 1 Jamia Millia Islamia, Centre for Physiotherapy and Rehabilitation Sciences - New Delhi - Delhi - India.; 2 An-Najah National University, Department of Applied Medical Sciences - Nablus - West Bank - Palestinian Territories.; 3 Independent Research Consultant, Independent Research Consultant - Toronto - Ontario - Canada.; 4 Somnogen Canada Inc., Somnogen Canada Inc. - Toronto - Ontario - Canada.

**Keywords:** Sleep, Postural Balance, Self-report, Universities, Students

## Abstract

**Objective:**

The primary aim was to investigate whether any association exists between poor sleep quality and deterioration in postural control among university student population.

**Materials and Methods:**

A case-control study was conducted in which sleep quality of 119 university students from different departments of Jamia Millia Islamia University, New Delhi, India was assessed using Pittsburgh sleep quality index (PSQI) following which the participants postural control, or dynamic balance was measured using the Y balance test (YBT). The participants were divided into two groups (A and B) based on their PSQI cut off scores. The YBT data was then evaluated for the dynamic balance assessment of the participants.

**Results:**

The mean age of the participants was 22.23±2.29 out of which 86 were female and 33 were male. The mean BMI of the participants was 21.58±3.66. Group A included 63 participants who had “good” sleep quality (global PSQI score < 5) whereas group B included 56 participants who had “poor” sleep quality (global PSQI score ≥ 5). Group comparisons based on t-test revealed a signiﬁcant difference (p<0.05) between means of the two groups, with the mean balance of group A being greater than that of group B. Also, chi-square testing showed no significant association between the BMI and dynamic balance scores for the participants (p<0.10).

**Conclusion:**

The findings of the study conclude that poor sleep quality is associated with a deterioration of postural control in university students. The study also revealed that there was no association between BMI and dynamic balance in this population.

## INTRODUCTION

Sleep is said to be “food for the brain”^1^. As per the Centers for Disease Control and Prevention (CDC) (2016)^2^, insufficient sleep is a growing public health concern that is inadequately recognized despite its ill effects on health. The outcomes of sleep problems, due to either inadequate sleep or an untreated sleep disorder can be serious. Major health outcomes ranging from cardiovascular diseases^3^ to psychological problems^4^ have been linked to poor sleep quality. Although most health outcomes related to poor sleep quality do not appear until later in life, it has been suggested that health outcomes may occur at an early stage, such as adolescence^5^.

The quality of sleep among university students has been examined extensively, with various studies concluding that the incidence of sleep disturbances is substantial, ranging from 19.2% to 57.5%^6,7^ being one of the most common health complaints among older adolescents and young adults^8^. The challenges of university life have often been singled out as the major contributor to this high level of poor-quality sleep^6^. It has been observed that university students, particularly those who suffer from poor academic performance, inadequate physical health, or mood disturbances, may in fact be suffering primarily from disturbed sleep^8^.

Among the major physical consequences of sleep disturbances, one is its effects on the maintenance of balance or postural control (PC) system. Dynamic balance is the ability to maintain postural stability and orientation with centre of mass over the base of support while the body parts are in motion^9^. Control of balance and posture are referred to as a complex ability, which is the result of interaction of various sensorimotor processes, and not merely a set of righting and equilibrium reflexes. These processes stabilize the body and maintain balance during dynamic work, and thus are critical for successful performance of basic movements and skills^10^.

There is also evidence that a close linkage exists between sleep and neural efficiency, one important component of which is the quality of PC. It has been found that experimentally induced sleep deprivation of 24 hours causes PC related problems that become exaggerated with the extent of the induced insomnia^11^. Sleep deprivation may have caused variations in sensory integrity, which in turn might have produced visual disturbances^12^. In another study, vibratory stimulation of the muscles during the sleep-deprived period caused a false impression of movement and was associated with a deterioration in body balance maintenance^13^. Many studies including youngsters have shown that PC is linked to sleep loss^11,14^, thus supporting the hypothesis that poor sleep hygiene may adversely impact the postural/balance control system. Although these studies are strongly suggestive of a close association between sleep quality and dynamic balance, the evidence on this point, particularly for adolescents and young adults, is still preliminary.

In addition, most of the research in this area has involved “acute” sleep deprivation as the main intervention, with few studies having considered the effects of long-term or chronic sleep disturbance on body balance. Previous experimentation on the effects of sleep impairment on PC have primarily focused on “acute” sleep deprivation rather than on more extended or chronic effects, and thus data on the impact of poor sleep quality on PC over a longer term are limited. The present study was carried out to investigate the probable adverse effects of sleep impairment in university students on the balance control system. The current study was therefore undertaken to address the need for a basic understanding of the relationship between sleep deprivation and PC. The study sought to test the hypothesis that impairment of sleep quality would negatively affect the dynamic balance of university students.

## MATERIALS AND METHODS

### Participants

The number of participants was determined using the G Power Software (Version 3.1.9.2). A sample size of 74 achieved 80% power; the correlation coefficient between the two variables was 0.60, with a level of significance of 0.05 based on a study done by Furtado et al.^15^. The sample size was increased to 119 participants to increase the power of the study. Students (day scholars) were recruited from different departments of Jamia Millia Islamia University, New Delhi, India. The participants’ age ranged from 18-28 years and included both genders. Participants were students from different departments who attended the university from 09:00 a.m. to 05:00 p.m. Potential study participants were excluded if they had any history of musculoskeletal disorders, an upper and/or lower extremity injury in the last 6 months, any neurological or cognitive disorders, visual/vestibular and somatosensory problems, the presence of any systemic disorder (e.g., rheumatological or diabetes mellitus), or use of any orthosis or prosthesis.

### Consent

Prior to participation, all research procedures were explained to participants and written informed consent was obtained in accordance with the Declaration of Helsinki. This study was approved by the Institutional Ethics Committee (IEC) of Jamia Millia Islamia University prior to its commencement. The study was also registered under the Clinical Trials Registry - India (Ref. No.: CTRI/2018/02/012041).

### Study protocol

**Figure 1:**
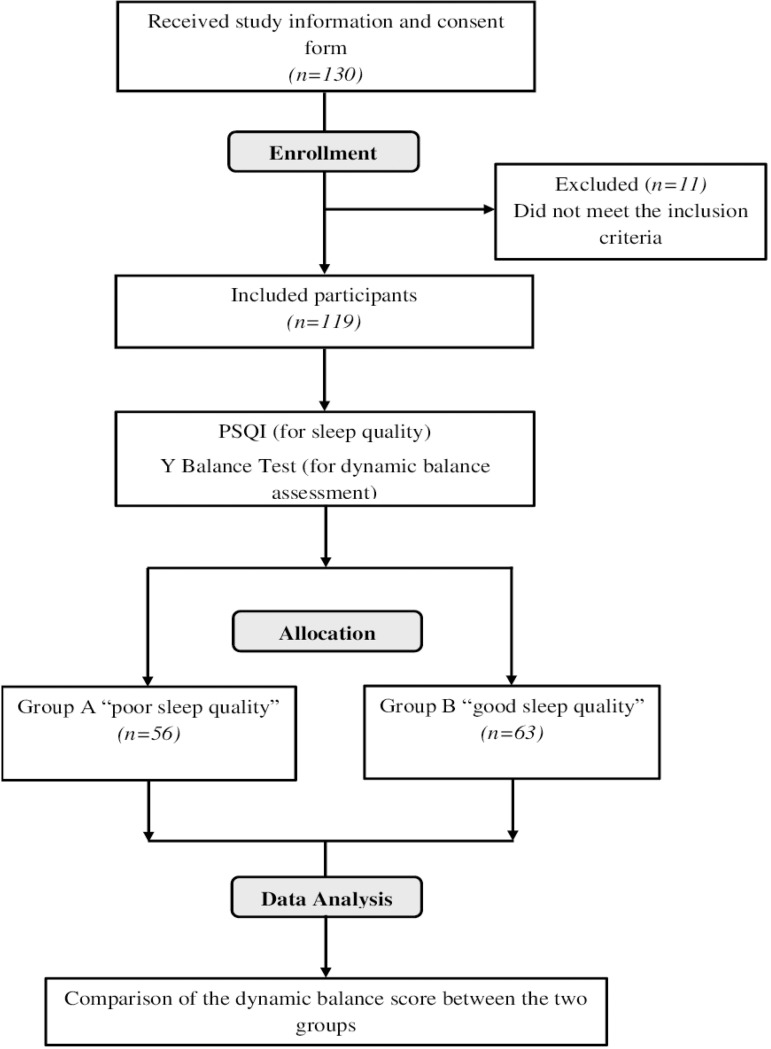
Study flowchart.

Following the basic demographic assessment, participants were required to fill out the Pittsburgh sleep quality index (PSQI) questionnaire, after which their balance measurements were taken using the YBT test. The complete procedure involved the filling out of the questionnaire and administration of dynamic balance assessment and took about 15-20 minutes for each participant. Participants were divided into two groups (group A: “poor” sleep quality; group B: “good” sleep quality) based on their global PSQI score (Figure 1).

### Sleep quality assessment

The Pittsburgh sleep quality index (PSQI) is a standard measure of sleep quality and the factors affecting it for a period of the past one month^16^. The questionnaire consists of 19 individual elements, which in turn produce seven components (subjective sleep quality, sleep latency, sleep duration, usual sleep efficiency, sleep disturbances, use of sleeping medication, and daytime dysfunction). The participants’ responses produce a global sleep quality score that ranges from 0-21. Separate components were not studied individually in this study.

All volunteers were given a simple demonstration plus general instructions for filling out the PSQI. This was done to minimize validity problems from exaggeration and/or under-reporting of the severity and/or frequency of symptoms. Participants who scored a global score of ≥5 were classified as having “poor” sleep quality, while those with a rating of <5 were classified as having “good” sleep quality.

### Dynamic balance assessment

**Figure 2:**
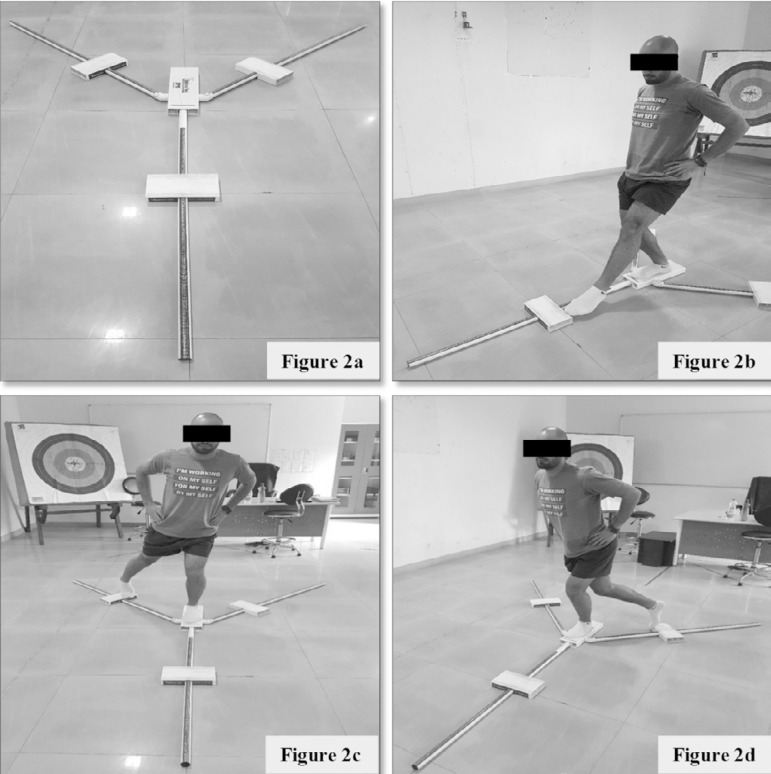
Y balance test: **a.** Y balance test kit; **b.** Anterior reach; **c.** Posteromedial reach; **d.** Posterolateral reach.

The Y balance test (YBT) was used for dynamic balance assessment (Figure 2a). The aim of the YBT is to measure an individual’s capacity to maintain balance on a single leg while extending the contralateral leg to its maximum^17^. The test is a modification of the star excursion balance test (SEBT) and similarly includes its three components, i.e., anterior, posteromedial, and posterolateral leg extensions^17^. It has a fixed platform with three PVC pipes connected in the front, posterolateral, and posteromedial directions. The rear rails are placed on 135 degrees of front rod, with 45 degrees between both the rear rails. Each bar is marked in centimeters to measure performance.

The YBT test was thoroughly explained and demonstrated to the participants. The participants practiced few trials before actual testing, with time limitations being applied to avoid excessive practice effects. The participants were tested directly after practice.

Participants stood on the centre platform with their right leg (which was tested first), with the toes just behind a red line, indicating the attempted position of the supporting leg on the platform. While maintaining a single-leg stance, the participants were asked to push the reaching indicator along a bar with the reaching foot in the three directions by simultaneously bending through the hip, knee, and ankle joints of the supporting leg, which was subject to dynamic stability testing.

When the participants reached their limit, they had to return to the starting position without pushing off or losing balance. To establish a consistent testing protocol and enhance the reproducibility of the test, the selected test sequence utilized the directional extensions “anterior (Figure 2b), posteromedial (Figure 2c) followed by posterolateral (Figure 2d), starting each direction with the right foot standing on the platform”^17^. Three trials were carried out for each direction. The participants were urged to try to reach an optimal distance. The successful completion of a trial required that the foot be positioned correctly behind the line and that all the minimum criteria for performing the action were met. The maximal reaching distance was measured by reading the measure on the bar at the edge (closer to participant) of the reaching indicator. Performance was not counted and repeated in the cases of: i) failure to return the reaching foot to starting position; ii) using the indicator as a support by placing the foot on top of it; iii) failure to keep the reach foot in touch with the indicator when it was in motion, either by kicking the reach indicator; and/or iv) failure in maintaining single leg stance, as when the reaching leg touched the floor or falling off the platform. A one-minute break was allowed before switching to the other leg.

To conclude the functional screening, lower limb length was measured in centimeters using a cloth tape measure for the analysis of the Y-balance scores. This was done with the participant lying in supine on an examination table and assessing the distance between anterior superior iliac spine and distal-most point of medial malleolus after squaring the pelvis.

To allow for comparisons, the reaching distance was normalized to limb length by dividing the reaching distance by limb length, and then multiplying the result by 100. Composite reaching distance was normalized by summing the three reaching direction distances and then dividing by three times the limb length, and then multiplying it by 100. A comparison of normalized composite scores to that of 94% of the limb length was used to group the dynamic balance of the participants into “normal” and “poor” balance categories^18^.

All the participants included in the present study were required to have normal or corrected to normal vision while performing the balance test; also, no participant had any kind of hearing problem. These requirements were established to rule out any alterations in the balance task performance due to vision or hearing impairment since balance is greatly weakened by a lack of “visual clues”^19^.

It is also known that PC is influenced by time-of-day. According to Bougard et al.^20^, “postural control was affected particularly in mid-day (10:00 a.m. and 2:00 p.m.) along with an improvement of postural control according to the time-of-day after a night of normal sleep”. In our study, balance assessment of all the participants was carried out during the same daytime hours, i.e., the middle of the day, to minimize any time-of-day effects.

### Statistical analysis

The data obtained from PSQI and YBT testing was analysed using IBM SPSS Statistics for Windows (Version 25.0. IBM Corp., Armonk, NY, USA). Chi-square tests were performed, and the *p* value was calculated to check for the existence of any association between the two main variables, i.e., sleep quality and dynamic balance. Spearman’s correlation coefficient between both variables was calculated to check the degree of association between them.

Significance testing using a *t*-test was used to compare means of the two groups (group A and group B). In addition, a chi-square test was carried out to check the association between body mass index (BMI) and dynamic balance. The significance level was set at *p*˂0.05 for the present study.

## RESULTS

All 119 participants underwent the full study procedures. The mean age of the study participants was 22.23±2.29 years, out of which 33 (27.7%) were males and 86 (72.3%) were females. The mean BMI of the study participants was 21.58±3.66, where 26 (21.8%) were underweight, 71 (59.7%) had normal weight, 19 (16.0%) were overweight and 3 (2.5%) were obese. Majority of the participants had right leg as the dominant leg 110 (92.4%) and only 9 (7.6%) had left leg as dominant leg (Table 1).

**Table 1.** Participants’ demographic characteristics, global PSQI scores, and YBT measurements.

		Total (%)^a^	Mean (SD)^b^
Age, years			22.23 (2.29)
Gender	Male	33 (27.7%)	
	Female	86 (72.3%)	
Height^c^, cm			161.92 (7.87)
Weight^d^, kg			56.50 (9.78)
BMI^e^, kg/m^2^			21.58 (3.66)
	Underweight	26 (21.8%)	
	Normal weight	71 (59.7%)	
	Overweight	19 (16.0%)	
	Obese	03 (2.5%)	
Dominant leg	Right	110 (92.4%)	
	Left	9 (7.6%)	
Global score of PSQI^f^	Good sleep quality	63 (52.9%)	2.74 (1.18)
	Poor sleep quality	56 (47.1%)	6.71 (1.11)
Anterior reach distance (N^g^)			69.72 (8.14)
Posterolateral reach distance (N^g^)			92.11 (11.38)
Posteromedial reach distance (N^g^)			96.89 (10.08)
Composite score (N^g^) 94% of limb length			86.24 (8.37)
			81.22 (4.81)
Composite score (N^g^) Vs. 94% of limb length	Normalized composite score more than 94% of limb length	82 (68.9%)	
	Normalized composite score ≤ 94% of limb length	37 (31.1%)	
Dynamic balance	Normal balance	82 (68.9%)	
	Poor balance	37 (31.1%)	

a Data are reported as number (percentage);

b Data are reported as mean (standard deviation);

c Height and Limb Length in centimeters;

d Weight in kilograms;

e Body mass index in kilogram per meter square;

f Pittsburgh sleep quality index;

g Normalized.

The analysis of the sleep quality showed that 63 (52.9%) had good sleep quality and 56 (47.1%) had poor sleep quality. The mean of global PSQI score for the study participants was 4.61±2.505. Mean of global PSQI score of good sleepers was 2.74±1.18 and for poor sleepers it was 6.71±1.11 as shown in Table 1.

The normalized values for YBT components were - anterior reach distance: 69.72±8.14; posterolateral reach distance: 92.11±11.38; and posteromedial reach distance: 96.89±10.08. The normalized composite score values came out to be 86.24±8.37 and the 94% of limb length was 81.22±4.81. Out of the total participants, 82 (68.9%) had normal balance and 37 (31.1%) had poor balance based on their YBT scores (Table 1).

The chi-square test was performed to check the association between sleep quality and dynamic balance. The test revealed that a statistically significant difference (*p*<0.05) existed, thus showing that sleep quality and dynamic balance were associated and dependent on each other. Chi-square testing for association between the BMI and dynamic balance revealed no significant difference between the two (*p*>0.05).

Spearman’s correlation coefficient was calculated to check the degree of association between the two study variables: sleep quality and dynamic balance. The correlation value turned out to be a ‘positive’ value of 0.240, thereby implying that sleep quality and dynamic balance move in the same direction. Following this, t-test was performed to check the significance of the correlation coefficient which indicated that correlation coefficient between both the variables in the population is significant (*p*<0.05). This revealed that participants with ‘good’ sleep quality had a good dynamic balance and vice versa.

The *t*-test for equality of means was performed to check whether there was any significant difference between the means of the two groups. Since the calculated *p* value was 0.009 (*p*<0.05), it was concluded that the mean balance of group A was greater than the mean balance of group B (Table 2). The results indicated that participants having a good sleep quality had better balance as compared to those having poor sleep quality.

**Table 2.** The t-test for equality of means between the two groups (group A and group B).

	Group A^a^ (n=63)	Group B^b^ (n=56)	
	Mean (SD)^c^	Mean (SD)	
	88.12 (7.47)	84.12 (8.87)	
	**t-test for equality of means**		
	**t**	**df**	**p-value**
Composite score (N^d^)	2.669	117	0.009

a Group A: “good” sleep quality;

b Group B: “poor” sleep quality;

c Data are reported as mean (standard deviation);

d Normalized.

## DISCUSSION

The results showed that 47.1% of the participants had poor sleep quality whereas 52.9% had good quality of sleep. The main outcome of the study demonstrated that there was a significant difference between the dynamic balance of the two groups revealing that the group of participants having a “good” sleep quality had better dynamic balance scores when compared to those having “poor” sleep quality. The findings, therefore, show that poor sleep quality adversely affects the performance of a significant motor task of the body, i.e., dynamic balance. The results of this work are supported by a recent study conducted by Furtado et al.^15^ which, to the best of our knowledge, is the only study that investigated the impact of “chronic” sleep deprivation on PC system. Their study involved the use of actigraphy and three questionnaires [Morningness - eveningness questionnaire (MEQ) from Horne and Östberg, the Pittsburgh sleep quality index (PSQI), and the Epworth sleepiness scale (ESS)] for assessment of sleep parameters. In contrast to the present study, postural assessment was done using Biodex balance system. The investigators concluded that “chronic” poor sleep quality impaired PC in a way like that of total sleep deprivation^15^.

Sleep is necessary for maintenance of optimal health, however, with a large number of people sleeping less than the suggested 8 hours per night, sleep restrictions have become prevalent in today’s society^1^, thereby causing variety of effects on motor functions, cognition, and mood^21^. It has been shown that sleep deprivation leads to deficits in various bodily functions^22^, which are specifically identified as disturbances in the circadian rhythms^15,23^, yet not much attention has been paid to the effects of deprivation of sleep on neuronal and muscular functions which are measured particularly via balance. Balance is one of the means of measuring the deficit of muscular and neurological performance connected to any head trauma, bone injury, muscle disorder, and balance disorders^24^. With ongoing researches, it has been observed that an adequate REM sleep phase is important for motor control because it has an impact on the proper regulation of muscle tonus^15^. Supporting this, many incidents like falls among vulnerable populations such as the elderly^25^ as well as driving accident incidents have been associated with various sleep disorders and the consequent deterioration of balance^26^. It has also been found that sleep deprivation of 24 hours causes PC related problems that might exaggerate with the insomnia^11^. The possible interpretation for this decline in PC due to impaired sleep were the variations in sensory integrity which in turn might be in sync with the sleep deprivation derived visual disturbances^12^.

The challenges of university life often cause students to adopt a severe prioritization of their activities. It has been observed that university students see adequate sleep as luxury, an attitude that is further reinforced by a general lack of awareness of the risks associated with inadequate sleep^27^. Older adolescents and young people often exhibit unhealthy sleep behaviour (e.g., sleep hygiene) that results in inadequate sleep at night as well as compensatory napping during the day^28,29^. It is also known that the stress associated with sleeplessness negatively affects the physical, mental, and emotional health of traditionally aged university students^30^. The failure of university students to get enough sleep is worrisome because sleep is a vital health behaviour with inevitable secondary effects on mental efficiency, academic performance, and ultimately on overall health^31^.

The human balance system is multifaceted and employs several mechanisms to integrate neural inputs for the larger purpose of preventing the human body from falling^32^^)^ and providing movement for upper and lower extremities. Balance is one of the major means of measuring the deficit of muscular and neurological performance.

The present study has provided evidence that sleep disturbance, i.e., self-related sleep quality is associated with a deterioration of PC, and thus may potentially be at the base of related performance decrements that have been linked to failures in neuromuscular regulation.

While the exact pathways and mechanisms of such related decrements remain to be studied, findings confirming their existence would certainly be consistent with the well documented evidence showing that, when compared to normal, night shift workers, or others suffering from inadequate sleep, exhibit a significantly greater rate of “slip and fall” injuries, industrial accidents, motor vehicle collisions, or other performance failures where motor coordination, and reaction times are critical^33^. It was difficult to compare our findings with other studies since most of the related studies were done for identifying the effect of 24-48 hours (short-term) of sleep deprivation on PC. We could not study the gender-based difference in our study due to a large imbalance between the number of female and male participants as the participation in the study was on a voluntary basis. Also, differences based on the side of the dominant limb (right or left) could not be predicted due to similar reasons. Another restriction of the study was different size of the groups, which was due to unknown sleep quality parameters when recruiting volunteers, as only after assessing sleep quality through PSQI, it was possible to determine the group to which a participant belonged.

The balance assessment in the present work was done using the YBT kit, which has been found to be an indicator of dynamic balance and injury prediction^18,34^. To the best of our knowledge, the YBT has not been used before to study the effect of impaired sleep on dynamic balance, however, SEBT had been used for assessing PC. Such tools provide an easy way for the balance assessment in clinical settings as compared to others which employ force platforms/plate and Biodex balance systems, the availability of which could be a significant issue in sleep clinics or other venues outside of physiotherapy diagnostic centres.

The study also tested for the association between BMI of the participants and their dynamic balance, but no significant association was found between the two variables. This might be because most of the participants (59.7%) had a normal BMI. However, according to a study done by Greve et al.^35^, it was observed that an increase in body mass was associated with increasing postural instability. Further studies are required to investigate the correlation between BMI and balance in the young adult population.

The integration of visual, vestibular, and proprioceptive inputs determines the PC^36^ and any impairment in sleep might lead to a change in this integration system. A possible cause for this may be attributed to the overlap of neurophysiological data during sleep deprivation and in the maintenance of the upright position, and also the diminished activation of the cortical areas involved in maintaining the upright position, cerebellum (cerebellar vermis efferent system) and the visual cortex^36-39^. Such results provide a rough mechanism behind the deterioration of balance due to poor sleep, however, no consistent and systematic evidence about the weaknesses of the main sub-mechanisms to control the balance during sleep deprivation has been found yet. Studies of middle-aged adults have shown that the impacts of sleep loss on PC seems to be related to external and internal factors such as cognitive status and sensory information integration^14^.

### Future research

The failure of university students who often struggle balancing between studies, work, and social commitments to get enough sleep is worrisome because sleep is a vital health behaviour linked to development, chronic health conditions, safety, and academic outcomes.

Further research among this population is needed including individuals with chronic sleep impairment considering gender, age, and dominant limb along with the execution of various tasks and activities under different sensory information conditions. Testing of PC should also be investigated further to assess its relevance for extending and confirming the validity of other tests of sleep quality as it provides an objective performance measure which may be carried out in contexts where it is desirable to screen large numbers of patients quickly. More elaborate testing using polysomnography or multimodal assessment of sleep (e.g., multiple questionnaires and actigraphy) could provide deeper knowledge.

## CONCLUSION

The present study demonstrates that poor sleep quality has a negative influence over the dynamic balance among university students. Those having poor sleep quality have poor balance as compared to those having a healthier sleep quality. Also, it was concluded that BMI is not associated with the dynamic balance in this population. Y balance test has potential for detecting impairment in dynamic balance in sleep-deprived individuals.
